# A pilot study on ecological momentary assessment in asylum-seeking children and adolescents resettled to Germany: Investigating compliance, post-migration factors, and the relation between daily mood, sleep patterns, and mental health

**DOI:** 10.1371/journal.pone.0246069

**Published:** 2021-02-01

**Authors:** Lauritz Rudolf Floribert Müller, Katharina Gossmann, Regina F. Schmid, Rita Rosner, Johanna Unterhitzenberger

**Affiliations:** Department of Psychology, Catholic University of Eichstätt-Ingolstadt, Eichstätt, Germany; University of Vienna, AUSTRIA

## Abstract

**Background:**

Asylum-seeking children and adolescents (ASCs) resettled to western countries show elevated levels of psychological distress. While research on the mental health of ASCs is increasing, less is known about their day-to-day living experiences such as their daily mood, sleep patterns, and post-migration factors. Moreover, no examination *in situ*, using smartphone-assisted ecological momentary assessment (EMA), has been conducted up to now among ASCs. Furthermore, we do not know if screening measures succeed in reflecting the daily mood of ASCs experienced in everyday life.

**Methods:**

We undertook a smartphone-assisted EMA study over a two-week period with 3 measurements a day. Participants were *N* = 40 ASCs from 10 different countries who had resettled to Germany. They completed standardized questionnaires screening for history of trauma and clinical symptoms (post-traumatic stress symptoms, depression, and anxiety) that were carried out in interview-like settings, and they participated in the subsequent EMA where they rated mood, sleep parameters, and post-migration factors on a daily basis. Multilevel models of clinical symptoms, daily mood, and sleep parameters were computed based on a total of 680 measurements.

**Results:**

The multiply traumatized and highly distressed participants reported different levels of discrimination, and various social activities and contacts in the EMA. The overall compliance rate was shown to be 40.5%. Higher PTSS and anxiety scores were associated with lower levels of daily mood and poorer outcomes of some sleep parameters. Depression scores were not associated with any of the variables assessed in the EMA.

**Conclusions:**

Smartphone-assisted EMA among ASCs resettled to Germany proved to be implementable despite a rather low compliance rate. Not only do ASCs show high symptom levels, they are also affected by these symptoms in their daily lives. The results emphasize the need for concise screenings and psychological treatment for this high-risk population. Limitations include the convenient nature of the sample and the lack of a comparison group.

## Introduction

Over the course of the years 2015 to 2017, at the peak of what became known as the European migrant crisis, almost 500,000 asylum-seeking children and adolescents (ASCs) under the age of 18 resettled to Germany [1–3]. Given the myriad of adversities that ASCs regularly experience in their home country and during different stages of flight [4,5], the screening of mental health problems of ASCs resettled to western countries has been a major focus of clinical research [6].

Whereas data on the rates of mental health problems of ASCs are appreciably increasing and becoming more accurate, less is known about the day-to-day experiences of ASCs with regard to domains such as post-migration factors, sleep patterns, and daily mood.

Moreover, most studies that focus on the everyday experiences of ASCs with regard to a variety of domains are of cross-sectional, more specifically retrospective, nature in the sense that participating ASCs were asked to rate their experiences over the course of a preceding time period (e.g. four weeks) at a given point in time (e.g. [7,8]). Naturally, this procedure is not specific to mental health research with ASCs but rather inherent to most non-experimental psychological research in and of itself [9]. However, this approach bears some considerations. In retrospective investigations, respondents might simply overlook past experiences, or these experiences might be conflated with current sensations and cognitions [10]. This can result in individuals recalling or rating their experiences differently from what they actually felt, thought, or perceived in a given situation, a phenomenon commonly known as recall or hindsight bias [11,12]. In fact, there is some evidence to suggest that memory inconsistencies among youths do appear regularly in psychological assessments, even with regard to severe experiences such as trauma [13]. For instance, in a 12-month follow-up study with ASCs in the Netherlands, 86.4% of the sample changed their response on at least one of the traumatic experiences assessed in the study [14]—which is largely in accordance with the proportion determined in studies with non-migration populations [15]. New technologies, like ecological momentary assessment (EMA), allow us to overcome some of these limitations. In EMAs, as opposed to conventional questionnaire assessments, participants respond *in situ* to the outcomes of interest using smartphones or comparable devices [16]. In doing so, respondents rate their present perceptions rather than evaluating what has happened in the past. In fact, studies that used the EMA approach have shown considerable discrepancy with retrospective examinations regarding the frequency with which certain symptoms were reported. For instance, in an EMA study with adult patients suffering from PTSD after childhood sexual abuse, participants reported roughly 50% more intrusions and flashbacks than in the subsequent retrospective investigation [17]. To date, however, little EMA research has been done among refugee youths. Although a study in Sweden examined intrusive memories in young refugees (age 16–25 years) in a daily diary format [18], real-time smartphone-based EMA among minor refugees has, to the authors’ knowledge, not yet been conducted. Some meta-analyses indicate that EMA studies among children and adolescents might yield lower compliance rates than studies among adults [19,20]. In addition, ASCs are likely to have lower language skills than native youths and may generally be less familiar with psychological test formats [21,22]—which is why we do not know whether the EMA approach is suitable for this population. Consequently, a key objective of this pilot study was to test the EMA approach among ASCs and to investigate their compliance to the EMA protocol as one aspect of its feasibility in this population.

Retrospective surveys indicate a wide range of post-migration factors that may affect the mental health of ASCs in the host country and suggest that ASCs regularly experience hassles regarding their asylum process [23,24], financial difficulties [23], and discriminatory incidents [7,8], among others. A very comprehensive review on risk and protective factors in ASCs distinguishes post-migration factors at the individual, family and community levels and demonstrates that lack of social support and resources in the host country and experiences of discrimination were among the most important post-migration factors to be associated with poorer mental health outcomes [25]. Ecological frameworks suggest that these post-migration factors are heavily interlinked [26], and, along with migration-related adversities, might have a cumulative effect on the mental health of ASCs [27]. Indeed, data from a Norwegian sample of minor refugees support this link by showing that higher levels of social support not only had a direct positive effect on mental health, but also had indirect positive effects on competence in dealing with experiences of discrimination [28]. However, the extent to which ASCs experience daily post-migration factors such as social support, resources, and discrimination is still unknown as assessed by real-time everyday data as these findings rely solely on retrospective investigations and there are, to date, no EMA investigations. In order to gather information on the most relevant aspects of the abovementioned post-migration factors in ASCs and as a precursor for a larger study that will look at the association between daily assessed post-migration factors and daily assessed mood and symptom scores of ASCs, this pilot study aimed at giving a first account of the as-is state regarding post-migration factors, daily mood, and sleep patterns.

The main body of clinical research among ASCs demonstrates large numbers of potentially traumatic experiences and high rates of psychological distress in this group, whereby posttraumatic stress symptoms (PTSS)/posttraumatic stress disorder (PTSD), depression, anxiety, and sleeping problems appear to be the major mental health problems [6,27,29,30]. In addition, the high rates of psychological distress seem to be stable over the course of the first years after arrival in the host country [31], even though a recent publication reported on the improvement of symptoms in a cohort of ASCs resettled to Germany [32]. However, it has been suggested very early on that ASCs might show good adaptation in daily life despite high symptom levels [33,34]. Primary caregivers might therefore simply overlook ASCs’ severe mental health status because it seems as though ASCs were not affected in everyday life. In addition, clinicians in low-resources environments, such as the refugee context, rely on concise screening measures that have mostly been validated in western countries [22]. While some may well be validated—or at least have been broadly used—with ASCs at the symptom level, cross-cultural validity of clinical screening measures remains an area of debate [35–37]. For example, we do not know whether they have predictive validity at a culturally less disputable, and therefore lower threshold and more easily retrievable, level in ASCs, that is, whether they succeed in reflecting negative consequences in everyday life as assessed by real-time data on areas such as daily mood and sleep problems. Even though we assume that standardized screening measures are likely to reflect these consequences, as this association has been documented in different settings [38–41], it is yet to be tested in the context of ASCs that, as mentioned above, may be a particular population where symptom levels might be high, yet, undetectable as demonstrated in everyday outcomes. Taken together, these results reveal the severe situation facing ASCs in western countries with regards to mental health issues, and the challenges for mental health care systems and professionals to adequately deliver services.

As outlined above, using EMA to inquire into the day-to-day experiences of ASCs, such as post-migration factors, daily mood, and sleep patterns, might constitute a worthwhile addition to the retrospective screening of ASCs’ symptom levels that has already been established in clinical research over the past few years. Accordingly, this pilot study aimed 1) to investigate the compliance of smartphone-assisted EMA among ASCs by deploying it for the first time in this group, 2) to depict the as-is state with regard to daily mood and post-migration factors of ASCs in day-to-day life (e.g. with whom do the ASCs interact most in everyday life? What activities do the ASCs pursue and enjoy in their daily lives? To what extent do they experience discrimination during the day?), and 3) to identify the association between ASCs’ symptom levels obtained by standardized questionnaire data and daily mood and sleep patterns obtained by EMA data. Specifically, we hypothesized that ASCs with higher symptom levels show poorer outcomes of daily mood and sleep parameters in everyday life.

## Methods

We followed the reporting guidelines for EMA studies as outlined recently by Trull and Ebner-Priemer [16,42] when drafting this manuscript.

### Ethics statement

The study was approved by the ethics board of the Catholic University Eichstätt-Ingolstadt in December 2016 (ethics approval number: 2016/23). An amendment for the original study was granted in July 2018 (ethics approval number: 2018/5). All participants and, if they were under 18, their legal guardians gave their written informed consent to participate in the study.

### Procedure

This study is an EMA study on ASCs’ experience of post-migration factors, and the association of daily assessed mood and sleep patterns with the mental health of this population reported in retrospective questionnaires. It was carried out in the summer of 2018 in Bavaria, Germany. It is based on two pre-studies. Study 1 [43] was a cross-sectional mental health screening among ASCs resettled to Germany conducted in 2017. Study 2 [32] was the 1-year follow-up of the initial screening. In this study, we report on the EMA subsequent to the 1-year follow-up, and link daily EMA data to standardized questionnaire data obtained from the 1-year follow-up.

**Fig 1:**
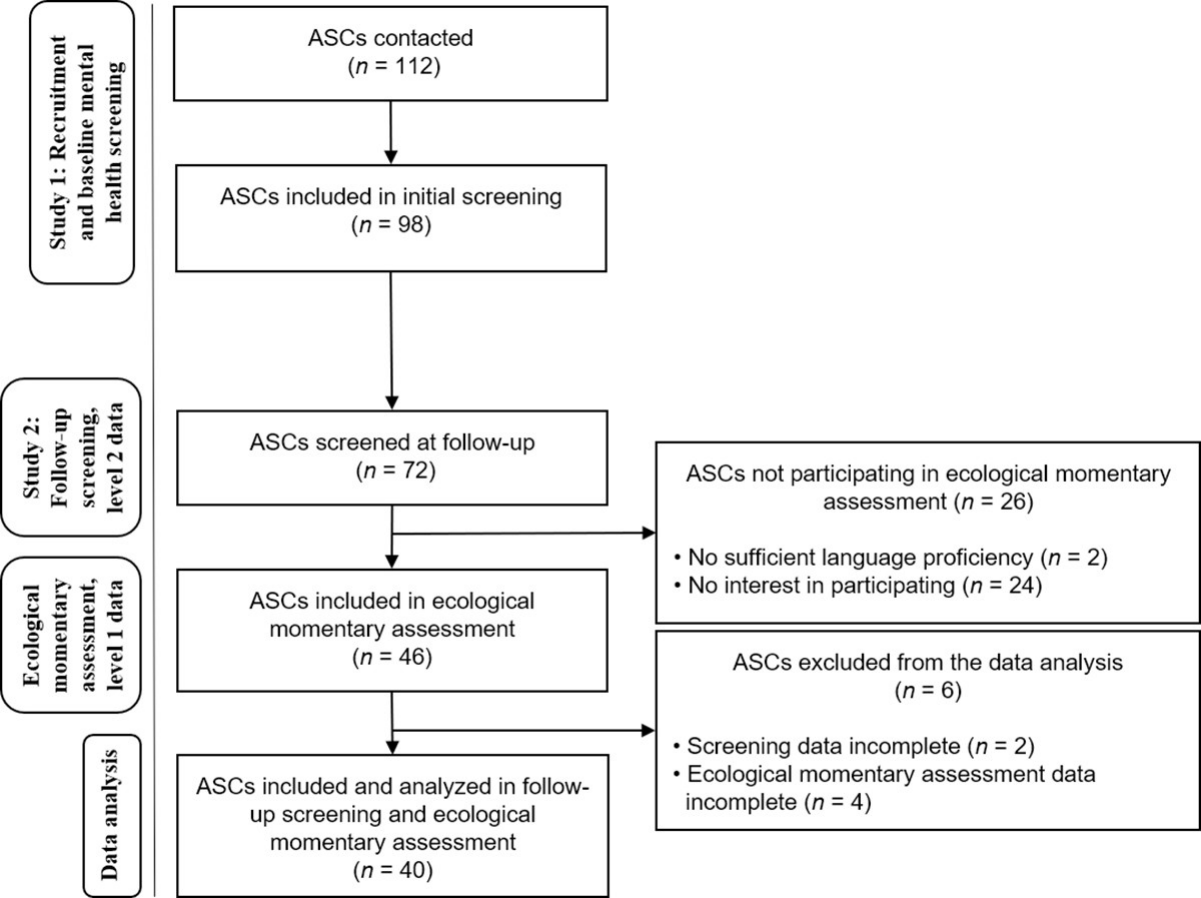
Participant flow chart. This chart shows the participant flow through different stages of study 1 (cross-sectional mental health screening), study 2 (1-year follow-up screenings), and this study (EMA).

Power analysis had been carried out prior to the baseline screenings in Study 1. According to the power analysis, a total of *N* = 112 ASCs had been recruited initially, 14 of whom had dropped out before completing the interviews, resulting in a sample of *N* = 98 ASCs for the baseline screening in Study 1. All youths, who had participated in baseline assessments, were contacted again in 2018. In this way, a sample of *N* = 72 ASCs could be secured for the 1-year follow-up assessment in Study 2. As part of the follow-up assessments in Study 2, all participants were asked whether they wanted to participate in the subsequent EMA investigation. Of these, 26 ASCs were not interested in participating in the EMA due to time issues or language difficulties, resulting in a total of *N* = 46 ASCs who agreed to participate in this study. Six participants were excluded from the analysis, due either to incomplete questionnaire data or incomplete EMA forms (completed prompts <3). Accordingly, this study comprises a final sample of *N* = 40 ASCs who completed both the 1-year follow-up questionnaires and the EMA, and were therefore included in the statistical analysis. Fig 1 shows the participant flow.

According to the inclusion criteria, ASCs who were under 18 when they entered the European Union and who had been resident in Germany for at least one year, were included in the original study (Study 1). At every stage of the different studies (baseline screening, follow-up, and EMA), participants and, in case of minors, their legal guardians were asked to give their informed written consent for participation. The follow-up screenings with standardized questionnaires (Study 2) were carried out in a face-to-face interview-like setting by either bachelor-level or master-level psychologists. See [32] for a more detailed account of the screening procedure.

After the face-to-face assessment in Study 2, all participating ASCs were asked if they were interested in taking part in the EMA study that took place over the course of two consecutive weeks. Nine days, on average, elapsed between the mental health screening and the completion of the first EMA prompt (*M* = 8.53, *SD* = 7.21, range 1–30). An additional inclusion criterion for the EMA was sufficient language proficiency in order to understand the forms. If the ASCs agreed to participate, they were given a programmed Android smartphone and were informed about the study aims, the procedure, the obligation of all professionals involved to preserve confidentiality, and the data privacy of the study. The authors clarified whether participants’ language proficiency was sufficient and went through all measures on an electronic tablet device prior to study intake so as to ensure the correct understanding of the items and the technical procedure. We designed the EMA as time-based monitoring with fixed-time sampling using the Android application movisensXS, version 1.4.8 (movisens GmbH, Karlsruhe, Germany). Since most participants still attended school, we chose fixed-time sampling in order to guarantee that no prompt was going to occur during school time or during scheduled afternoon activities. Throughout the day, three prompts occurred over a period of two consecutive weeks, in the morning at 7.15 a.m., in the afternoon at 2.15 p.m., and in the evening at 9.15 p.m. Participants could choose to suspend any prompt for up to 25 minutes. If participants did not respond to a prompt, it re-occurred 3 times with a time lag of 5 minutes before being counted as a missing. In order to enhance compliance, participants could contact the researchers at any time if they experienced any technical issues or problems of comprehension. In case of any mental or physical distress caused by the assessment, the researchers provided immediate psychological support. However, no emergencies occurred in the context of this study. At the end of the EMA, participants were given EUR 10, and the researchers retrieved the smartphones and extracted the EMA data.

### Measures

#### Standardized questionnaires

The sociodemographic characteristics were assessed by self-report at the beginning of the face-to-face assessment. Asylum status, placement changes, educational background in the country of origin, and previous psychosocial treatment were covered in addition to common demographic variables. Furthermore, the following measures were conducted in German during the interviews.

The *Child and Adolescent Trauma Screen* (CATS; [44]) consists of three parts. It assesses potentially traumatic events (CATS trauma list) and the frequency of PTSS during the previous two weeks (CATS symptom scale). The CATS trauma list originally comprises 15 items, and we added 4 items that examine potentially traumatic events related to flight and resettlement experiences (deprivation, dangerous journey, imprisonment and/or abduction, and the committing of violating acts (voluntary or involuntary)). In the CATS symptom scale, the participants rated the frequency of PTSS over the course of the previous two weeks on a 4-point Likert scale. The CATS symptom scale consists of 20 items and its sum score ranges from 0 to 60 with a suggested cut-off value of 21 to indicate clinically significant levels of PTSS. The CATS is based on the DSM-5 criteria for PTSD and has shown good psychometric properties [45]. It has previously been used among the ASC population in a variety of studies [32,46,47]. In this study, the inter-item reliability of the CATS symptom scale was *α* = .81.

The *Hopkins Symptom Checklist-37 for Adolescents* (HSCL-37A; [48]) examines both internalizing symptoms such as depression and anxiety, and externalizing symptoms. We used the included subscales for anxiety (range 10–40, cut-off 20) and depression (range 15–60, cut-off 33) as they represent two major symptom clusters among ASCs [27]. The frequency of the symptoms was assessed over the previous four weeks on a 4-point Likert scale. The HSCL-37A is a widely used questionnaire and has shown good psychometric properties for different populations including ASCs. The inter-item reliability of the constituent scales in this study was as follows: depression (15 items, *α* = .87), anxiety (10 items, *α* = .76).

#### Ecological momentary assessment (EMA)

The German version of the *Multidimensional Mood Questionnaire* (MDMQ; [49]) is a questionnaire on daily mood that, in its long version, comprises 24 items which are rated on a 6-point Likert scale. Analogous to Wilhelm & Schoebi [50], we modified the questionnaire for the EMA in order to guarantee an appropriate length and tailor it to the language proficiency of the ASCs. Consequently, the modified MDMQ version used in this study included 6 bipolar items rated by a visual analogue scale ranging from 0 to 100 where respondents were asked to indicate how they felt at that given moment. The scales, each comprising 2 bipolar items, were as follows: good mood vs. bad mood, awake vs. tired, calm vs. nervous. The bipolar items of the scales used during the EMA were happy ↔ unhappy and content ↔ discontent for the *mood scale*, alert ↔ tired and rested ↔ worn out for the *tiredness scale*, and relaxed ↔ tense and calm ↔ nervous for the *nervousness scale*. The term “very” was added to the endpoints of the visual analogue scales, and “0” represented the “positive” endpoints (i.e. happy/content, alert/rested, and relaxed/calm) and “100” represented the “negative” endpoints (i.e. unhappy/discontent, tired/worn out, tense/nervous) of the scales. Beyond that, no other linguistic anchor points were added. The MDMQ is a widely used questionnaire and has already been utilized in EMA studies with a variety of populations [51] including adolescents [52,53]. Within-person inter-item reliability for the mentioned scales in this study was as follows, good mood vs. bad mood (2 items, *α* = .84), awake vs. tired (2 items, *α* = .72), and calm vs. nervous (2 items, *α* = .74).

Since many ASCs present sleep problems [29,30], we assessed sleep functioning as additional aspects by quality of sleep, sleep duration, and sleep onset latency (i.e., time needed to fall asleep). Participants rated sleep quality using a single item on a visual analogue scale ranging from 0 (“very well”) to 100 (“very badly”), with lower numbers representing a more “positive” outcome. Furthermore, they rated sleep duration and sleep onset latency by stating hours and minutes. These items were addressed exclusively in the mornings. We designed both daily mood (MDMQ) and sleep parameters forms as forced-choice items.

In addition, the EMA examined everyday experiences regarding post-migration factors, namely activities undertaken by the participants and their valence (as aspects of everyday resources), the occurrence of social interactions and their valence (as aspects of everyday social support), and experiences of discrimination. Participants recorded activities they had undertaken since the last prompt in a single multiple-choice item and rated whether they experienced them as pleasant or unpleasant on a bipolar visual analogue scale ranging from 0 to 100 where lower numbers represented a less pleasant specification of the item (with “very pleasant” and “very unpleasant” as the linguistic endpoints). The same procedure was applied to social interactions, where participants could first indicate with whom they had contact since the last prompt. Participants could then state the person with whom they had the most memorable interaction, and were asked to rate the valence of this interaction on a bipolar visual analogue scale. Furthermore, experiences of discrimination were examined using a modified 4-item version of the Everyday Discrimination Scale (EDS) [54] adjusted to the EMA design, such that participants could state how often they had experienced different discriminatory events since the last prompt (i.e., ‘being treated with less courtesy or respect than youths without history of flight’; ‘people acting as if they thought I was not as smart as youths without history of flight’; ‘people acting as if they were afraid of me; ‘being threatened or harassed’). The operationalization of post-migration factors and sleep patterns as well as their item formulation were based on consensual literature research pointing to the most relevant aspects of these constructs [25,55,56] and consultation with experts in the field. See S2 Table for the German EMA forms used in this study, and see S3 Table for the translated forms.

### Statistical analyses

Data were analyzed using SPSS statistics, version 25. Prior to analysis, the uncentered questionnaires and EMA data were analyzed using descriptive statistics. Next, all person-related predictors (level 2) were grand-mean centered [57] in line with the recommendations by Ohly, Sonnentag, Niessen, and Zapf [58]. We then performed random intercept models to examine whether PTSS, depression or anxiety (level 2) were predictors of the mean levels of daily mood (good mood vs. bad mood, awake vs. tired, calm vs. nervous) and sleep parameters (quality, duration, and onset latency) (level 1). Based on the hierarchical structure of data, in which the daily within-person variables (daily mood and sleep parameters) were nested in persons, we performed random intercept models for each level 1 outcome variable [57]. Based on the null models we calculated the intraclass correlations (*ICC*) in order to estimate the distribution of variances for all level 1 variables. To test our hypotheses, all level 2 variables including main effects (PTSS, depression, anxiety) and interaction effects (PTSS × depression, PTSS × anxiety, depression × anxiety) were added simultaneously. The models included a random intercept. For the model fit, we used chi-square tests to examine the differences in the log likelihood ratios and degrees of freedom between the null models and the main and interaction models.

## Results

### Sample characteristics

Forty ASCs were included in the final analysis. The majority were male (*n* = 34, 85%), members of the Islamic faith (*n* = 34, 85%), had immigrated unaccompanied to Europe (*n* = 17, 43%), came from Afghanistan (*n* = 20, 50%), and attended a public school in Germany (*n* = 27, 68%). Most of the adolescents kept in touch with family members who were living in their home country at the time of the study (*n* = 35, 88%). The majority of ASCs were living in either semi- (*n* = 14, 35%) or full-care facilities (*n* = 11, 28%) of the German child and youth welfare system or were living with their families in private accommodation (*n* = 11, 28%). The asylum applications of most participating ASCs had been accepted (*n* = 28, 70%), even though the applications of some participants had been rejected (*n* = 11, 28%) or were still pending (*n* = 1, 2%). The majority had not received any kind of psychotherapeutic treatment (*n* = 32, 80%) since their arrival in Germany, 5 ASCs had completed psychotherapy (12%), and 3 participants were receiving some kind of therapeutic treatment at the time of assessment (8%). The mean age of the participating ASCs was 17.5 years (*SD* = 1.88, range 13 to 20 years) and they had been living in Germany for almost 3 years (*M* = 2.92, *SD* = .85). No differences regarding the sociodemographic characteristics between the ASCs, who participated in the EMA and the ones who did not, were identified using *t*-tests for independent samples with all *p* values above .07.

Participating ASCs had experienced a mean of almost 9 different potentially traumatic events in their lifetime (*M* = 8.7, *SD* = 3.35). The proportion of individuals who scored above the clinical cut-offs was as follows, CATS symptom score (*n* = 21, 52.5%), HSCL-37A total scale (*n* = 11, 27.5%), HSCL-37A depression scale (*n* = 11, 27.5%), and HSCL-37A anxiety scale (*n* = 9, 22.5%). Participants in EMAs differed significantly from non-participants with respect to mental health outcomes, such that participants showed higher levels of PTSS (CATS symptom score, *t*(70) = 2.44; *p* < .05), depression (HSCL-37A depression scale, *t*(70) = 2.94; *p* < .01), and anxiety (HSCL-37A anxiety scale, *t*(70) = 2.13; *p* < .05). See S1 Table for further information regarding demographics and mental health outcomes assessed using standardized questionnaire data.

#### Compliance with the EMA protocol

To allow for a sufficient intra-person variance in EMA measurement points, only participants who responded to a minimum of 3 prompts were included in further analyses. Overall, 42 EMA prompts occurred for each participant over the course of the study, resulting in a total of 1680 prompts across all participants and measurement points. Of those, participants dismissed a total of 123 prompts (7.3%), and responded to a total of 680 prompts, 57 of which were incomplete (8.4%), resulting in an overall compliance rate of 40.5%. Compliance rates were highly heterogenous ranging from 7.1% to 100% between persons, and participants responded to an average of *M* = 17 prompts (*SD* = 9.85, range 3–42). The measurement point with the highest compliance was the evening prompt (48.8%), compliance rates in the morning and in the afternoon were 43.9% and 28.8%, respectively. Compliance was not correlated with any of the symptom levels assessed in the study as identified by correlation analyses with all *p* values above .31. Participants responded within 2.5 minutes, on average (*SD* = 6.18), after the first prompt occurred and took 2 minutes to answer the EMA questionnaire (*M* = 2.0, *SD* = 2.4).

#### EMA data on daily mood, sleep parameters, and post-migration factors

Table 1 gives the descriptive statistics of the EMA data with regard to daily mood and sleep parameters. Participants reported different levels of daily mood over the course of the day, ranging from *M* = 41.9 (*SD* = 25.3) to *M* = 39.81 (*SD* = 28.07) for the mood scale, from *M* = 51.11 (*SD* = 26.27) to *M* = 43.12 (*SD* = 27.99) for the tiredness scale, and from *M* = 40.28 (*SD* = 24.31) to *M* = 38.92 (*SD* = 26.3) for the nervousness scale. Their reported mean level of sleep quality was *M* = 45.28 (*SD* = 23.41), and they reported an average of some 5 hours of sleep a night (*M* = 5.42, *SD* = 2.55), and a latency of one hour, on average, to fall asleep (*M* = 1.2, *SD* = 1.06).

**Table 1:** Descriptive representation of daily mood (good mood vs. bad mood, awake vs. tired, calm vs. nervous) and sleep parameters (quality, duration, onset latency) as reported by the participants (*N*_*1*_ = 40) at different points during the EMA (*N*_*2*_ = 680).

	Morning ^a^		Afternoon ^a^		Evening ^a^	
Variables	*M* (*SD*)	range	*M* (*SD*)	range	*M* (*SD*)	range
Daily mood, *M* (*SD*)						
good mood vs. bad mood ^b^	41.9 (25.3)	0–99.5	39.81 (28.07)	0–100	40 (28.6)	0–100
awake vs. tired ^b^	51.11 (26.27)	1–100	44.26 (27.2)	0–100	43.12 (27.99)	0–100
calm vs. nervous ^b^	40.28 (24.31)	0–100	38.92 (26.3)	0–100	39.5 (27.21)	0–100
Sleep parameters, *M* (*SD*)						
quality ^b^	45.28 (23.41)	0–100	–	–	–	–
duration in hours	5.42 (2.55)	0–12	–	–	–	–
onset latency in hours	1.2 (1.06)	0–4	–	–	–	–

^a^ Across all participants and prompts.

^b^ Assessed using a visual analogue scale whereby lower numbers represent more positive values, and higher numbers represent more negative values.

Table 2 gives the descriptive statistics of the EMA data with regard to post-migration factors. For social interactions, either virtually or in person, friends from the same country were indicated most frequently as the most memorable social interactions (in the evening, *n* = 71, 27.6%; in the afternoon, *n* = 33, 22.1%). In both cases this implied that the social interaction had occurred since the last prompt. Siblings were indicated the least frequently as the most memorable interactions both in the afternoon (*n* = 8, 5.4%) and in the evening (*n* = 17, 6.6%). Participants rated their most memorable social interactions similarly in the evening (*M* = 29.53, *SD* = 25.77) and in the afternoon (*M* = 27.58, *SD* = 25.29). Social interactions with fathers were rated as most pleasant both in the evening (*M* = 9.22, *SD* = 7.95) and in the afternoon (*M* = 14.21, *SD* = 14.39).

**Table 2:** Descriptive representation of post-migration factors (social interactions, activities undertaken, discrimination) as reported by the participants (*N*_*1*_ = 40) at different points during the EMA (*N*_*2*_ = 435).

	Afternoon		Evening	
Variables	Total occurrences ^a^ *n* (%)	Valence ^b^ *M (SD)*	Total occurrences ^a^ *n* (%)	Valence ^b^ *M (SD)*
Most memorable social interaction	149	27.58 (25.29)	257	29.53 (25.77)
Mother	19 (12.8)	20.32 (27.55)	50 (19.5)	18.14 (20.67)
Father	20 (13.4)	14.21 (14.39)	18 (7.0)	9.22 (7.95)
Siblings	8 (5.4)	22.5 (17.97)	17 (6.6)	21.65 (25.34)
Other relatives	11 (7.4)	45.7 (23.25)	20 (7.8)	38.4 (23.93)
Friends from my home country	33 (22.1)	25.73 (21.39)	71 (27.6)	38.72 (25.38)
German friends	24 (16.1)	28.33 (25.79)	24 (9.3)	30.87 (27.94)
Professionals (e.g. caregivers)	18 (12.1)	46 (30.97)	18 (7.0)	33.17 (24.26)
Another person	16 (10.7)	26.37 (24.7)	39 (15.2)	32.85 (28.32)
Activities undertaken	–	–	256	
Sport	–	–	98 (38.3)	19.63 (21.57)
Religious practices (e.g. praying)	–	–	52 (20.3)	13.75 (14.2)
Listening to music or watching a movie	–	–	128 (50.0)	28.45 (24.84)
Educational activities (e.g. reading, doing homework)	–	–	62 (24.2)	27.37 (22.95)
Another activity	–	–	67 (26.2)	28.51 (25.06)
Discrimination–Being treated with less courtesy or respect than youths without history of flight	149		260	
No such incident	130 (87.2)		217 (83.5)	
At least one incident	19 (12.7)		43 (16.5)	
Discrimination–People acting as if they thought I was not as smart as youths without history of flight	149		260	
No such incident	136 (91.3)		234 (90.0)	
At least one incident	13 (8.7)		26 (10.0)	
Discrimination–People acting as if they were afraid of me	149		260	
No such incident	143 (96.0)		249 (95.8)	
At least one incident	6 (4.0)		11 (4.2)	
Discrimination–Being threatened or harassed	149		260	
No such incident	144 (96.6)		244 (93.8)	
At least one incident	5 (3.4)		16 (6.2)	

*Note*. Post-migration factors were queried in the afternoon and in the evening only which is why morning prompts are not displayed.

^a^ Across all participants and prompts.

^b^ Assessed using a visual analogue scale whereby lower numbers represent more positive values, and higher numbers represent more negative values.

Activities undertaken by the participants were reported in the evening only since most ASCs were attending school. The most frequent activity reported by the participants was using media such as listening to music or watching videos (*n* = 128, 50%) which was rated *M* = 28.45 (*SD* = 24.84) on the valence scale. Religious activities were the rarest activities (*n* = 52, 20.3%), but were perceived the most pleasant (*M* = 13.75, *SD* = 14.2).

Across all prompts, participants reported 139 incidents of at least one discriminatory event since the last prompt (8.5% of all queries), and a mean of some 5 discriminatory events over the course of the study (*M* = 4.88, *SD* = 7.72). ‘Being treated with less courtesy or respect’ was the most common incident both in the afternoon (*n* = 19) and in the evening (*n* = 43), whereas ‘being threatened or harassed’ was the rarest incident in the afternoon (*n* = 5), and ‘people acting as if they were afraid of me was the rarest incident in the evening (*n* = 11).

### Relation between symptom levels assessed using standardized questionnaire data and daily mood and sleep parameters assessed using EMA data

*ICCs* indicated that a considerable proportion of variance could be observed on the within-person level: *ρ* = .46 for good mood vs. bad mood; *ρ* = .35 for awake vs. tired; *ρ* = .47 for calm vs. nervous; *ρ* = .37 for quality of sleep; *ρ* = .49 for duration of sleep; and *ρ* = .49 for sleep onset latency. For the model fit, we examined the differences in the log likelihood ratios and degrees of freedom between the null models and the main and interaction models by chi-square tests. As stated in Table 3, none of the chi-square tests for good vs. bad mood, χ^2^(6) = 9.68, *p* = .139, awake vs. tired, χ^2^(6) = 8.62, *p* = .196, and calm vs. nervous, χ^2^(6) = 7.58, *p* = .271, proved to be significant, indicating no significant difference between the null models and the main and interaction models. For sleep parameters (see Table 4), differences between the null models and the main and interaction models proved to be insignificant for quality, χ^2^(6) = 2.15, *p* = .906, and highly significant for duration, χ^2^(6) = 18.61, *p* < .01), and onset latency, χ^2^(6) = 36.28, *p* < .001.

**Table 3:** Multilevel models predicting daily mood (good mood vs. bad mood, awake vs. tired, calm vs. nervous).

	Good vs. bad mood		Awake vs. tired		Calm vs. nervous	
Fixed effects	Estimate (*SE*)	*p*	Estimate (*SE*)	*p*	Estimate (*SE*)	*p*
Intercept	35.61 (4.84)	< .001**	44.33 (4.30)	< .001**	34.09 (4.85)	< .001**
PTSS	1.23 (.64)	.032*	.86 (.57)	.070	1.30 (.64)	.025*
Depression	-1.14 (.96)	.124	-.74 (.86)	.199	-1.23 (.96)	.105
Anxiety	2.65 (1.31)	.026*	2.5 (1.16)	.020*	2.46 (1.31)	.035*
PTSS × depression	.16 (.11)	.153	.12 (.09)	.212	.19 (.11)	.089
PTSS × anxiety	-.34 (.20)	.092	-.21 (.17)	.231	-.37 (.19)	.066
Depression × anxiety	.13 (.13)	.306	-.01 (.11)	.944	.10 (.13)	.413
Random effects	Estimate (*SE*)		Estimate (*SE*)		Estimate (*SE*)	
Intercept	271.56 (79.92)		192.88 (63.94)		263.56 (74.73)	
Model comparison	Main effects and interaction model	Null model	Main effects and interaction model	Null model	Main effects and interaction model	Null model
–2* Log likelihood	5881.30	5890.98	5995.76	6004.38	5684.90	5692.48
χ^2^	9.68		8.62		7.58	
*df*	6		6		6	

Note. *N*_*1*_ (persons) = 40, *N*_*2*_ (assessments) = 680.

*** *p* < .001

** *p* < .01

* *p* < .05.

**Table 4:** Multilevel models predicting sleep parameters (quality, duration, onset latency).

	Quality		Duration		Onset latency	
Fixed effects	Estimate (*SE*)	*p*	Estimate (*SE*)	*p*	Estimate (*SE*)	*p*
Intercept	43.72 (4.99)	< .001**	6.15 (.53)	< .001**	1.25 (.25)	< .001**
PTSS	1.02 (.64)	.062	-.15 (.07)	.021*	.01 (.03)	.427
Depression	-.65 (1.02)	.202	.09 (.11)	.197	-.04 (.05)	.224
Anxiety	1.18 (1.39)	.266	.23 (.15)	.067	.13 (.07)	.039*
PTSS × depression	.10 (.11)	.354	-.01 (.01)	.347	.00 (.01)	.727
PTSS × anxiety	-.178 (.20)	.375	.02 (.02)	.352	-.00 (.01)	.824
Depression × anxiety	.01 (.13)	.961	-.03 (.01)	.072	-.00 (.01)	.693
Random effects	Estimate (*SE*)		Estimate (*SE*)		Estimate (*SE*)	
Intercept	206.51 (77.55)		2.48 (.87)		.61 (.19)	
Model comparison	Main effects and interaction model	Null model	Main effects and interaction model	Null model	Main effects and interaction model	Null model
–2* Log likelihood	2001.60	2003.75	1012.78	994.16	625.86	589.58
χ^2^	2.15		18.62**		36.28***	
*df*	6		6		6	

Note. *N*_*1*_ (persons) = 40, *N*_*2*_ (assessments) = 680.

*** *p* < .001

** *p* < .01

* *p* < .05.

The results show that some symptom measures assessed in the standardized questionnaires significantly determined the mean level of daily mood (see Table 3). Higher levels of PTSS were significantly associated with higher levels of bad mood (γ *=* 1.23, *t*(31) = 1.925, *p <* .05). The same was true for anxiety (γ = 2.65, *t*(32) = 2.021, *p* < .05), indicating that ASCs with higher ratings of PTSS and anxiety showed higher levels of bad mood. Anxiety was the only variable related to tiredness (γ = 2.50, *t*(30) = 2.16, *p* < .05), such that ASCs with higher levels of anxiety showed higher levels of tiredness. Both anxiety and PTSS, were significantly related to nervousness (PTSS, γ = 1.30, *t*(34) = 2.04, *p* < .05; anxiety, γ = 2.46, *t*(35) = 1.877, *p* < .05), such that ASCs suffering from higher symptom levels reported higher levels of nervousness. Neither depression scores nor any of the analyzed interactions between the level 2 variables proved to be associated with any of the level 1 outcome measures.

For sleep parameters, we observed some level 2 effects (see Table 4). None of the between-person symptom scales turned out to be significantly associated with sleep quality. For sleep duration, there was a negative association with PTSS (γ = -.15, *t*(31) = -2.12, *p* < .05), showing that participants with higher levels of PTSS slept less. Anxiety levels were significantly related to sleep onset latency (γ = .13, *t*(37) = 1.82, *p* < .05), such that ASCs with higher ratings for anxiety needed more time to fall asleep.

## Discussion

To our knowledge, this is the first study to report on a smartphone-assisted EMA study undertaken among ASCs resettled to western countries. ASCs are a psychologically distressed [6,27] and hard-to-reach population that appears to be understudied with regard to domains such as day-to-day living, daily mood, and post-migration factors [6].

Consequently, one major aim of this pilot study was to probe the EMA approach in this particular group, and examine participants’ compliance to the EMA protocol, in order to gain insight as to whether it might constitute a worthwhile addition to conventional assessment formats for ASCs. With this in mind, the results of this study are promising although some limitations have to be borne in mind. The approach proved to be implementable overall given that a large number of the ASCs contacted prior to the study showed interest in participating in the assessments, and the survey format appeared to appeal overall to the participants. Electronic devices such as smartphones are widely used and appreciated among refugee populations [59], and arguably even more so in refugee youth. This may have facilitated the favourable outcome regarding the readiness of ASCs to participate in this particular form of assessment. Having said that, the compliance rate in this study (40.5%) was rather low compared to a literature review on EMA studies in children and adolescents where rates ranged from 54.6% to 96.21% [20]. This finding corresponds to a recent EMA study with adult refugees [60] where the overall compliance rate was shown to be 55% after excluding 7 participants with a particularly low response rate (<20%). There are several plausible explanations for the low compliance rate determined in our study. First, we excluded only those participants who completed less than 3 prompts (<5%), whereas exclusion criteria in other EMA studies among adolescents vary broadly and are sometimes as high as <50% [61]. Consequently, excluding more participants with especially low response rates would have resulted in a more favourable overall compliance rate. Second, refugee youths might not be familiar with psychological survey formats [21,22,36,62]. This is why problems of comprehension might have emerged over the study runtime, possibly resulting in a lack of motivation to pursue the study protocol. Even though we ensured correct understanding of all items prior to study intake, and offered close guidance and support over the course of the study, we cannot dispel this uncertainty. Third, the sampling frequency might have affected the compliance rate. While there seems to be an association between fewer prompts per day and higher compliance rates in non-clinical samples, quite the opposite proved to be the case in clinical samples as determined in a meta-analysis of EMA studies with children and adolescents [20]. Even though our sample was not a clinical sample per se, participants were severely traumatized and showed high levels of psychological distress that clearly exceed the levels of psychological problems reported in non-clinical community settings [63,64]. Following the line of reasoning of the Wen et al. (2017) meta-analysis [20], these severely distressed individuals may experience the daily assessment of symptom-related aspects such as daily mood as meaningful and intrinsically relevant. Accordingly, close-meshed monitoring including more prompts per day while, at the same time, taking into account the multitude of obligations of ASCs over the course of the day, might produce more favourable compliance outcomes. To sum up, future EMA investigations with ASCs should, first, carefully weigh the ideal frequency and times of day to incorporate assessment prompts. Second, they might consider including compliance-linked incentives as well as ASCs’ caregivers to guide adherence to the study protocol. Last, they should consider adding a conclusive interview to gather information regarding participants’ acceptance of the survey format and the question why some participants respond only infrequently. This could shed light on further aspects of the feasibility of EMA studies among ASCs, and could help to identify potential design modifications that might facilitate better compliance.

The second objective of this study was to give an account of the day-to-day experiences of ASCs after resettlement with regard to post-migration factors and daily mood. In view of the pilot character of this study, it must first be pointed out that a central shortcoming of this study is the lack of a comparison group. Including an appropriate comparison group (e.g., native-born adolescents, young migrants without a history of flight, or the comparison of unaccompanied to accompanied ASCs) would allow for more in-depth statistical and clinical conclusions. Moreover, there are no corresponding normative values for either the age group under investigation or the modified version of the MDMQ [65]—the same is true for the further measures used in this study. Interpretations and classifications of the participants' data should therefore be made with caution. Since ASCs are a little studied population, it is nevertheless worthwhile to give a first picture of daily collected mood and post-migration factors and to draw at least some tentative comparisons to methodologically similar studies with non-ASCs.

Participants reported moderate levels of daily mood over the course of the day, with the morning prompt showing the poorest levels of daily mood on all scales recorded (good vs. bad mood, awake vs. tired, calm vs. nervous). This could be a consequence of their mediocre rating of sleep quality [66] and very short sleep duration (some 5.4 hours) as compared to both a sample of ASCs in the UK [29] and a sample of German adolescents [67] who both reported some 7–8 hours of sleep on weekdays. This—in conjunction with the fact that the American Academy of Sleep Medicine recommends 8 to 10 hours of sleep per night in the 13–18 age group [56]—further illustrates that sleep problems are central symptoms of ASCs [30]. With regard to the levels of daily mood assessed in this study, there is only a limited number of comparable EMA studies that used both the modified version of the MDMQ and examined adolescents. For example, compared to a study with adolescents suffering from non-suicidal self-injury in Germany, participants in our study showed somewhat higher mean daily mood values which, on the other hand, were also well below the values of the healthy control group [52]. This might be another indication of the severe levels of psychological distress in our sample as determined by retrospective clinical screening measures.

Across all prompts, interactions with friends from the same country were reported most frequently and were considered pleasant overall. These social networks appear to be important sources of social support for ASCs [68]. This might have been encouraged by the fact that most participants were still integrated in full-care units of the German child and youth welfare system where they regularly live alongside youths from the same country. Even tough interactions with their parents occurred more rarely; they were consistently rated to be the most pleasant and supportive interactions, in particular the ones with their father. The latter is a notable finding considering that past research has mainly outlined the essential role of the mother as a protective factor [69,70]. As many unaccompanied ASCs in this study stayed in touch with their parents over the phone, this re-emphasizes the fact that access to electronic communication devices is absolutely key for them to experience social support from their parents [68]. Accordingly, ASCs who had difficulty staying in touch with their relatives presented poorer psychological functioning in earlier studies [71]. With regard to recreation activities, participants reported doing sports and using multimedia (e.g. listening to music or watching videos) most frequently and rated them as pleasant overall. Of all activities queried in the assessments, religious practices such as praying were reported the least frequently, yet, rated the most pleasant. This points to the value of religious practices as potential coping strategies for ASCs who have resettled to western countries [72–74]. Doing sports also ranked among the most pleasant activities and this is largely in line with past research that has highlighted the vital role of sports for ASCs after resettlement. This may impact a wide array of favourable processes such as acculturating into a host country [75,76]. Consequently, special sports programs for refugee youth have been developed and evaluated in the past [77], and encouraging their proliferation and accessibility to ASC is desirable. By and large, the reported experiences of discrimination in our study were comparable to a daily diary study of ethnic minority youth in the US, with participants in our study reporting roughly one event more over the course of the study [78]. However, the comparability is limited as there was a different measure in use. As was reported there, one of the negative effects of the discrimination experiences was that already anxious individuals showed further increased anxiety values over the course of the days. Negative effects of experiences of discrimination are also extensively documented in ASC literature [25]. Consequently, the prevention of such experiences should be a decisive goal of current policies.

Finally, we sought to investigate the relationship between ASCs’ symptom levels obtained using standardized questionnaires and daily mood and sleep parameters obtained using EMAs. The results indicate that higher symptom levels (i.e., PTSS and anxiety scores) were associated with poorer daily mood (i.e., bad mood, tiredness, nervousness) and poorer outcomes of some sleep parameters (i.e., duration and onset latency) in everyday life. While this might be considered a corollary of high symptom levels, given that these mental health problems regularly encompass sleeping problems and low mood [79], it is still an important finding and can be thought of as a further component of the predictive validation of the CATS and HSCL-37A [80]. Symptom levels reported in retrospective investigations and as assessed using brief screening measures did, in fact, correspond to daily assessed implications and were shown to affect the participating ASCs in their day-to-day lives. It has been argued that querying lower-threshold problems, such as sleep parameters or mood, could be a worthwhile and culturally more accepted form of symptom retrieval in primary care settings that could still provide initial indications of clinically noticeable symptoms [29], and the findings of our study support this argumentation. Besides that, sleeping problems and poor daily mood have been shown to have detrimental effects on important domains such as academic performance and self-esteem [81–83]. Consequently, and in order to facilitate integration and good everyday functioning, providing care models that include systematic screening and access to treatment of mental health problems should be a major aim of current policies. Despite the paucity of adequate treatments, there are promising approaches that have been pursued in the past [46,47,84]. It is, moreover, worth noting that participants who took part in the EMA, showed significantly higher symptom levels than those who merely completed the standardized questionnaires. This indicates that ASCs with the highest levels of psychological distress showed particular interest in participating in the EMA. While this may be contrary to our presumptions, it illustrates both the need of severely traumatized ASCs for appropriate care and their openness to long-term monitoring [85], and increases the scope for the potential of innovative technology-based treatment approaches.

### Limitations

Certain limitations should be borne in mind with respect to the appraisal of this study. As has been discussed above, the major shortcomings of this study are the lack of a control group and the relatively low compliance rate. Beyond that, it should be pointed out that our sample was of a convenient nature for various reasons. The participants were not recruited at random, and only those who had acquired sufficient language proficiency in order to understand the EMA forms were included in the study which might have resulted in selection bias. Also, the sample size was rather small which impeded more nuanced analyses and limits generalizability to the population of ASCs as a whole. Apart from issues concerning the sample composition, there may also have been procedural distortions. Even though we offered close guidance and support to all participants throughout the study runtime, the compliance rate was quite low which may impair the validity of the current findings. As has been discussed earlier, future investigations should, therefore, take certain steps to address this shortcoming. Furthermore, we cannot exclude with absolute certainty that problems of understanding cropped up over the course of the EMAs which might have resulted in invalid responses to the questionnaires. Finally, it should be mentioned that we only assessed post-migration factors and daily mood but not symptom levels on a daily basis. Gathering further data on day-to-day symptom levels might, therefore, contribute to deriving more in-depth conclusions about the mental health of this vulnerable population.

### Conclusions

Examining everyday daily mood and post-migration factors in ASCs from 10 different countries using smartphone-assisted EMA was shown to be implementable. Not only do ASCs show high symptom levels, they are also affected by these symptoms in their daily lives as determined by the EMA data. We would welcome the regular implementation of EMA in future research efforts with ASCs. In order to ensure access for the widest possible range of participants, including ASCs with a low language level or no literacy, possible modifications should include translated EMA forms or audio-assisted assessment tools [86]. Due to the lack of evidence pointing to empirically supported strategies to promote compliance in children and adolescents [20,87], we can only assume that individually tailored assessment schedules, compliance-linked incentives, and the involvement of ASCs’ caregivers could offer an opportunity for improvement. Future research efforts could also focus on the relationship between daily mood and post-migration factors as well as intra-individual fluctuations during the day. The results of this study, as well as the EMA approach in and of itself, may help to establish low-threshold screening possibilities in primary care and to identify specific resources and coping strategies of ASCs in everyday life that should be promoted accordingly in the future.

## Supporting information

S1 Table Socio-demographic characteristics of the participating ASCs. (DOCX)

S2 Table Original wording of the items used for the scales *post-migration factors* (social contacts, activities undertaken, experiences of discrimination) and *sleep parameters* in the ecological momentary assessment. (DOCX)

S3 Table English translation of the items used for the scales *post-migration factors* (social contacts, activities undertaken, experiences of discrimination) and *sleep parameters* in the ecological momentary assessment. (DOCX)

## Abbreviations

ASC: Asylum-seeking children and adolescents
CATS: Child and Adolescent Trauma Screen
EDS: Everyday Discrimination Scale
EMA: Ecological momentary assessment
ERSS: Everyday Resources and Stressors Scale
HSCL-37A: Hopkins Symptom Checklist-37 for Adolescents
ICC: Intraclass correlation
L1 (variable): Level 1 (variable)
L2 (variable): Level 2 (variable)
PTSD: Posttraumatic stress disorder
PTSS: Posttraumatic stress symptoms
