# Supplementary material for: A pilot study on ecological momentary assessment in asylum-seeking children and adolescents resettled to Germany: Investigating compliance, post-migration factors, and the relation between daily mood, sleep patterns, and mental health
Source: PLoS One. 2021 Feb 1;16(2):e0246069. doi: 10.1371/journal.pone.0246069 (PMC7850498; doi:10.1371/journal.pone.0246069)
Supplement: S1 Table — (DOCX) [file pone.0246069.s001.docx]

**S1 Table. Socio-demographic characteristics and mental health outcomes of the participating ASCs.**

| **S1 Table. Socio-demographic characteristics and mental health outcomes of the participating ASCs (*N* = 40).** | |
| --- | --- |
| Age in years, *M* (*SD*) | 17.5 (1.88) |
| Gender, *n* (%) |  |
| male | 34 (85) |
| female | 6 (15) |
| Country of origin, *n* (%) |  |
| Afghanistan | 20 (50.0) |
| Iraq | 5 (12.5) |
| Syria | 4 (10) |
| Eritrea | 3 (7.5) |
| Gambia | 3 (7.5) |
| others | 5 (12.5) |
| Length of stay in months, *M* (*SD*) | 35.05 (10.4) |
| Asylum status, *n* (%) |  |
| accepted | 28 (70) |
| rejected | 11 (27.5) |
| pending | 1 (2.5) |
| Family status in Germany, *n* (%) |  |
| unaccompanied | 17 (42.5) |
| accompanied by both parents | 8 (20) |
| accompanied by mother | 4 (10) |
| accompanied by father | 1 (2.5) |
| accompanied by adult siblings | 6 (15) |
| accompanied by other relatives | 3 (7.5) |
| missing values | 1 (2.5) |
| Mental health outcomes, *M* (*SD*) |  |
| CATS-TL | 8.7 (3.35) |
| CATS-SS | 19.57 (9.23) |
| HSCL-Dep | 29.23 (8.52) |
| HSCL-Anx | 17.23 (4.26) |
| Note. *CATS-TL* = CATS trauma list; *CATS-SS* = CATS PTSS scale; *HSCL-Dep* = HSCL-37A depression scale; *HSCL-Anx* = HSCL-37A anxiety scale. | |
