# Supplementary material for: A pilot study on ecological momentary assessment in asylum-seeking children and adolescents resettled to Germany: Investigating compliance, post-migration factors, and the relation between daily mood, sleep patterns, and mental health
Source: PLoS One. 2021 Feb 1;16(2):e0246069. doi: 10.1371/journal.pone.0246069 (PMC7850498; doi:10.1371/journal.pone.0246069)
Supplement: S2 Table — (DOCX) [file pone.0246069.s002.docx]

**S2 Table. Original wording of the items used for the scales *post-migration factors* (social contacts, activities undertaken, experiences of discrimination) and *sleep parameters* in the Ecological Momentary Assessment.**

| **S2 Table. Original wording of the items used for the scales *post-migration factors* (social contacts, activities undertaken) and *sleep parameters* in the Ecological Momentary Assessment.** | | |
| --- | --- | --- |
| Scale | Items |  |
| Post-migration factors |  |  |
| social interactions (SI) | SI1. Mit welchen Personen hattest du in den letzten Stunden persönlich oder digital (z. B. Handy) Kontakt?  SI1.A Meine Mutter  SI1.B Mein Vater  SI1.C Meine Geschwister  SI1.D Weitere Verwandte  SI1.E Freunde, die aus demselben Land kommen wie ich  SI1.F Deutsche Freunde  SI1.G Professionelle, z. B. Lehrer, Betreuer usw.  SI1.H Jemand anderes | Multiple choice, forced choice |
|  | SI2. An welchen Kontakt erinnerst du dich noch besonders? Mit wem war dieser Kontakt?  SI2.A Meine Mutter  SI2.B Mein Vater  SI2.C Meine Geschwister  SI2.D Weitere Verwandte  SI2.E Freunde, die aus demselben Land kommen wie ich  SI2.F Deutsche Freunde  SI2.G Professionelle, z. B. Lehrer, Betreuer usw.  SI2.H Jemand anderes | Single choice, forced choice |
|  | SI3. Wer war diese Person? | Displayed if “SC2.H” was marked, open response format |
|  | SI4. Wie war der Kontakt mit dieser Person?  SI4.A angenehm ↔ unangenehm  SI4.B unterstützend ↔ nicht unterstützend  SI4.C entspannt ↔ angespannt  SI4.D freundlich ↔ aggressiv | Visual analogue scale (0 to 100), forced choice |
| activities undertaken (AU) | AU1. Was hast du in den letzten Stunden gemacht?  AU1.A Sport  AU1.B Religiöse Aktivitäten, z. B. Beten  AU1.C Musik hören, Filme/Videos anschauen  AU1.D Etwas für die Schule/Arbeit, z. B. Hausaufgaben, Lesen  AU1.E Eine andere Aktivität | Multiple choice, forced choice |
|  | AU2. Wie fandest du diese Aktivität?  AU2.Activity 1 angenehm ↔ unangenehm  AU2.Activity 2 angenehm ↔ unangenehm | Displayed all activities marked in “AU1”, visual analogue scale (0 to 100), forced choice |
| Sleep parameters |  |  |
| duration (SD) | SD1. Wie viele Stunden hast du heute Nacht ungefähr geschlafen?  SD1.A 0 Stunden ↔ 14 Stunden | Visual analogue scale (0 to 100), forced choice |
| onset latency (SOL) | SOL1. Wie lange hast du ungefähr gebraucht, um einzuschlafen?  SOL1.A 0 Stunden ↔ 4 Stunden | Visual analogue scale (0 to 100), forced choice |
| quality (SQ) | SQ1. Wie hast du heute geschlafen?  SQ1.A sehr schlecht ↔ sehr gut | Visual analogue scale (0 to 100), forced choice |
