# Supplementary material for: A pilot study on ecological momentary assessment in asylum-seeking children and adolescents resettled to Germany: Investigating compliance, post-migration factors, and the relation between daily mood, sleep patterns, and mental health
Source: PLoS One. 2021 Feb 1;16(2):e0246069. doi: 10.1371/journal.pone.0246069 (PMC7850498; doi:10.1371/journal.pone.0246069)
Supplement: S3 Table — (DOCX) [file pone.0246069.s003.docx]

**S3 Table. English translation of the items used for the scales *post-migration factors* (social contacts, activities undertaken, experiences of discrimination) and *sleep parameters* in the Ecological Momentary Assessment.**

| **S3 Table. English translation of the items used for the scales *post-migration factors* (social contacts, activities undertaken) and *sleep parameters* in the Ecological Momentary Assessment.** | | |
| --- | --- | --- |
| Scale | Items |  |
| Post-migration factors |  |  |
| social interactions (SI) | SI1. With whom did you have personal or digital (e.g. cell phone) contact in the last hours?  SI1.A My mother  SI1.B My father  SI1.C My siblings  SI1.D Other relatives  SI1.E Friends from the same country  SI1.F German friends  SI1.G Professionals, e. g. teachers, caregivers  SI1.H Someone else | Multiple choice, forced choice |
|  | SI2. Which contact do you remember most? With whom was this contact?  SI2.A My mother  SI2.B My father  SI2.C My siblings  SI2.D Other relatives  SI2.E Friends from the same country  SI2.F German friends  SI2.G Professionals, e. g. teachers, caregivers  SI2.H Someone else | Single choice, forced choice |
|  | SI3. Who was this person? | Displayed if “SC2.H” was marked, open response format |
|  | SI4. How was the contact with this person?  SI4.A pleasant ↔ unpleasant  SI4.B supportive ↔ not supportive  SI4.C relaxed ↔ tense  SI4.D friendly ↔ aggressive | Visual analogue scale (0 to 100), forced choice |
| activities undertaken (AU) | AU1. What have you been doing in the last hours?  AU1.A Sports  AU1.B Religious activities, e. g. praying  AU1.C Listening to music, watching movies or videos  AU1.D Something for school/work, e. g. homework, reading  AU1.E Another activity | Multiple choice, forced choice |
|  | AU2. How did you find this activity?  AU2.Activity 1 pleasant ↔ unpleasant  AU2.Activity 2 pleasant ↔ unpleasant | Displayed all activities marked in “AU1”, visual analogue scale (0 to 100), forced choice |
| Sleep parameters |  |  |
| duration (SD) | SD1. How many hours did you sleep tonight approximately?  SD1.A 0 hours ↔ 14 hours | Visual analogue scale, forced choice |
| onset latency (SOL) | SOL1. How long did it take you to fall asleep?  SOL1.A 0 hours ↔ 4 hours | Visual analogue scale, forced choice |
| quality (SQ) | SQ1. How did you sleep tonight?  SQ1.A very well ↔ very badly | Visual analogue scale (0 to 100), forced choice |
